# Controlling topological entanglement in engineered protein hydrogels with a variety of thiol coupling chemistries

**DOI:** 10.3389/fchem.2014.00023

**Published:** 2014-05-14

**Authors:** Shengchang Tang, Bradley D. Olsen

**Affiliations:** Department of Chemical Engineering, Massachusetts Institute of TechnologyCambridge, MA, USA

**Keywords:** engineered protein hydrogels, coiled-coil, thiol-X click chemistries, entanglement, branching

## Abstract

Topological entanglements between polymer chains are achieved in associating protein hydrogels through the synthesis of high molecular weight proteins *via* chain extension using a variety of thiol coupling chemistries, including disulfide formation, thiol-maleimide, thiol-bromomaleimide and thiol-ene. Coupling of cysteines via disulfide formation results in the most pronounced entanglement effect in hydrogels, while other chemistries provide versatile means of changing the extent of entanglement, achieving faster chain extension, and providing a facile method of controlling the network hierarchy and incorporating stimuli responsivities. The addition of trifunctional coupling agents causes incomplete crosslinking and introduces branching architecture to the protein molecules. The high-frequency plateau modulus and the entanglement plateau modulus can be tuned by changing the ratio of difunctional chain extender to the trifunctional branching unit. Therefore, these chain extension reactions show promise in delicately controlling the relaxation and mechanical properties of engineered protein hydrogels in ways that complement their design through genetic engineering.

## Introduction

Control of advanced mechanical properties of hydrogels is central to their applications. In many situations, such as engineering simulant materials of articular cartilage or blood vessels (Drury and Mooney, 2003), hydrogels are required to support mechanical load and maintain structural integrity. Multiple approaches have been developed to address this issue, with emphasis on increasing the network toughness and elastic moduli, such as preparing double-networks (Gong et al., 2003; Sun et al., 2012) and fabricating composite materials (Haraguchi and Takehisa, 2002; Wang et al., 2010a). In some scenarios where dynamic properties such as injectability, self-healing, shape memory, and controlled degradation/reinforcement are desirable, non-covalent crosslinks and stimuli-responsive triggers can be incorporated into gels to fulfill the application requirements (Kloxin et al., 2009; Holten-Andersen et al., 2011; Guvendiren et al., 2012; Glassman et al., 2013). However, many methods developed in synthetic polymer systems cannot be readily applied to protein hydrogels, and manipulating the mechanical response of the gels presents a new challenge. Because the function of a protein is related to its hierarchical structure and the diversity of amino acid functional groups, site-specific and orthogonal reactions are often required to preserve protein properties in the final gel. In addition, the modification reactions can usually only be performed in aqueous buffers due to the poor solubility of proteins in organic solvents.

Currently there are two main strategies for chemically modifying proteins to manipulate the mechanical properties of protein hydrogels. The first strategy is covalently crosslinking protein polymer chains by adding crosslinking reagents (Trabbic-Carlson et al., 2003; Li et al., 2011; Chung et al., 2012), catalysts (Shen et al., 2005; Lv et al., 2010) and/or enzymes (Davis et al., 2010). As this method only modifies the amino acid residues participating in junction formation, the protein strands can retain most of their function (e.g., elasticity and stimuli responsivity). In addition, many mechanical properties at equilibrium, such as modulus and maximum swelling ratio, can be controlled by varying the dosage of the crosslinking agents according to well-known laws of hydrogel physics and network theories (Graessley, 2004; Tanaka, 2011; Kim et al., 2013). In a typical crosslinking reaction, however, the formation of network imperfections, such as dangling chains and inelastic loops, is usually uncontrollable and difficult to quantify (Zhou et al., 2012). The second strategy is coupling synthetic polymers and proteins to prepare chimeric copolymers (Jing et al., 2008; Sahin and Kiick, 2009; Wu et al., 2011; Glassman and Olsen, 2013; Glassman et al., 2013). Interactions leading to self-assembly of the polymer or protein component then create nanostructure within the hydrogel, which in some cases can be triggered by external stimuli. This method allows delicate manipulation of hydrogel mechanics, such as erosion rate, toughness and elasticity. However, preparing hybrid hydrogels adds challenges in materials synthesis and purification.

Recently, we developed a facile method for introducing entanglements into protein hydrogels through simple chain extension reactions, and we exploited this entanglement effect to engineer new mechanical responses into the materials (Tang et al., 2014). This strategy minimally modifies the protein molecules, only extending protein chains by establishing disulfide end linkages, yet it creates a drastic enhancement in many mechanical properties, including the low-frequency modulus, resistance to creep, extensibility and toughness. The disulfide linkages are redox responsive, which provides opportunities to regulate network mechanics by redox stimuli.

In addition to disulfide coupling, several other thiol chemistries are promising candidates for coupling high molar mass proteins: thiol-maleimide, thiol-bromomaleimide and thiol-ene coupling. Thiol-maleimide chemistry enables site-specific modification of cysteine residues and has been a popular route for constructing protein-based bioconjugates (Canalle et al., 2010; Stephanopoulos and Francis, 2011). Recently, the thiol-maleimide addition has been found to be reversible, offering new opportunities to control the degradation of the conjugates (Baldwin and Kiick, 2011). Thiol-dibromomaleimide conjugation has emerged as another important “click” chemistry (Smith et al., 2010; Jones et al., 2011; Robin et al., 2013), but only recently has its potential in the chain extension reaction been appreciated (Cui et al., 2013). The thiomaleimide adduct is redox responsive: the addition of reducing reagents such as β-mercaptoethanol (BME) reverses the reaction and recovers the unmodified proteins. Compared to other coupling chemistries, the thiol-ene reaction is the most rapid coupling method, with extremely high bimolecular rate constants in the thiyl-alkene addition, ranging from 10^5^ to 10^7^ M^−1^ s^−1^ (Northrop and Coffey, 2012), which is 4–5 orders of magnitude larger than thiol-maleimide addition (Schelté et al., 1999). Despite potential side reactions (Schöneich, 2008), there exist many successful examples demonstrating the use of thiol-ene chemistry in direct protein modification (Dondoni et al., 2009; Weinrich et al., 2010; Valkevich et al., 2012).

In this work, thiol-maleimide, thiol-bromomaleimide and thiol-ene coupling chemistries for application in protein chain extension reactions to produce entangled hydrogels are compared. In addition, the ability to use chain extension points for the modification of proteins with poly(*N*-isopropylacrylamide) (PNIPAM) side chains is established, demonstrating thermally responsive mechanical behavior. By using trifunctional chain coupling agents, branched proteins are also prepared, and the effect of the ratio of di to trifunctional chain coupling agent on entanglements is assessed. These experiments demonstrate sophisticated control of molecular structure and network mechanics in engineered protein hydrogels, yielding the ability to control chain topology, chain entanglement, and chemical functionalization all through thiol-based chain coupling chemistries.

## Materials and methods

### Materials

Bismaleimide diethylene glycol (**1a**) was purchased from Thermo Fischer. Maleimide-PEG(1k)-maleimide (**1b**) was purchased from creative PEGWorks. The average molecular weight of the PEG portion determined by ^1^H NMR was 1082 Da. β-cyclodextrin (βCD) and 2,2'-Azobis[2-(2-imidazolin-2-yl)propane]dihydrochloride (VA-044) were purchased from Wako USA. All other chemical reagents were purchased from commercial sources (Sigma-Aldrich and VWR) and used as received unless otherwise noted.

### Characterization

^1^H NMR spectra were recorded in CDCl_3_ or DMSO-*d*_*6*_) using a Varian Mercury 300 MHz Spectrometer in Department of Chemistry Instrumentation Facilities at MIT. High-resolution mass spectrometry (HRMS) data was obtained on a Bruker Daltonics APEXIV 4.7 Tesla Fourier Transform Ion Cyclotron Resonance Mass Spectrometer. Matrix assisted laser desorption ionization mass spectrometry (MALDI) data was obtain on a Bruker Omniflex MALDI-TOF Mass Spectrometer. α-Cyano-4-hydroxycinnamic acid (CHCA) was used as matrix. Gel permeation chromatography (GPC) was performed on an Agilent 1260 system equipped with a Wyatt Optilab T-rEX refractive index (RI) detector and a Wyatt Mini-DAWN multi-angle light scattering (LS) detector. The mobile phase was DMF supplemented with 0.02 M LiBr and the instrument was operated at 1.0 mL/min at 70°C.

### Protein Cys-P_4_-Cys expression and purification

Protein expression and purification by ammonium sulfate purification have been described previously (Tang et al., 2014). In this study, the proteins were additionally purified by anion exchange chromatography using a HiTrap Q Sepharose HP 5 mL column (GE healthcare, WI), eluting with a gradient of 0–500 mM NaCl in 6 M urea and 20 mM Tris (pH 8.0). A typical isolation yield is 120 mg per liter culture and the protein purity was determined to be >97% by sodium dodecyl sulfate-polyacrylamide gel electrophoresis (SDS-PAGE).

### Chemical compound synthesis

#### Dibromomaleimide-alkyne

The synthesis procedure reported elsewhere (Jones et al., 2011) was slightly modified. Potassium carbonate (0.89 g, 6.50 mmol) was suspended in 20 mL acetone and 2,3-dibromomaleimide (1.5 g, 5.90 mmol) was added to the slurry in one portion and the reaction was left to stir at room temperature for 5 min. Propargyl bromide (80% in toluene, 0.72 mL, 6.50 mmol) was added dropwise to the mixture over 10 min. After 24 h, solvent was removed under vacuum and the mixture was redissolved in DCM. Salts were filtered and the residue was loaded onto a silica gel column vacuum. The crude product was purified by flash chromatography, eluted with 0–2% MeOH in DCM (TLC *R*_*f*_ = 0.78, stained with KMnO_4_ solution) to afford 492 mg **4** as a white powder (yield 28.5%).

^1^H NMR (300 MHz, CDCl_3_) δ 4.38 (d, *J* = 2.4 Hz, 2H), 4.83 (t, *J* = 2.4 Hz, 1H). DART HRMS (m/z) calcd for C_7_H_4_Br_2_NO_2_ [M+H]^+^: 293.8586; found 293.8585.

#### Dibromomaleimide-β-CD

To a 25 mL Schlenk tube was added **4** (58.5 mg, 0.20 mmol), mono-6-deoxy-6-azido-β-cyclodextrin (Petter et al., 1990) (116.0 mg, 0.10 mmol), Cu(I)Br (14.3 mg, 0.10 mmol) and 5 mL DMF. The mixture was degassed through 3 freeze-pump-thaw cycles. 2,2'-Bipyridine (15.6 mg, 0.10 mmol) was added to the frozen mixture and the mixture was degassed one more time. The click reaction was performed at 30°C for 24 h. Catalyst was removed by passing through a short alumina column and the product was obtained by precipitation in acetone twice to get 63 mg **2** as a yellow powder (yield 43.4%).

^1^H NMR (300 MHz, DMSO-*d*_*6*_) δ 8.06 (s, 1H), 5.95–5.60 (m, 14H), 5.10–4.67 (m, 9H), 4.65–4.42 (m, 6H), 3.79–3.48 (m, 28H), 3.43–3.21 (m, overlaps with HOD). MALDI-TOF MS (m/z) calcd for C_49_H_72_Br_2_N_4_O_3_Na [M+Na]^+^: 1473.22; found 1473.22.

#### Bisallyl tetraethylene glycol

Tetraethylene glycol (5.0 g, 25.7 mmol) was dissolved in 30 mL anhydrous DMF. Sodium hydride (3.5 g, 60% in mineral oil, 87.5 mmol) was added to the mixture in one portion. The mixture was cooled with a water bath and allowed to stir for 30 min at room temperature. After the reaction stopped bubbling, allyl bromide (9.5 g, 78.4 mmol) was added dropwise. After 14 h, excess NaH was quenched by adding 10 mL saturated NH_4_Cl aqueous solution. The mixture was diluted with EtOAc, washed with DI water and brine, and dried over MgSO_4_. Then solvent was removed under reduced pressure. The crude product was purified by silica gel column chromatography, eluted with 50% EtOAc in hexanes (TLC *R*_*f*_ = 0.41, stained with KMnO_4_ solution) to afford 3.65 g **3** as a slightly yellow liquid (yield 51.8%).

^1^H NMR (300 MHz, CDCl_3_) δ 5.91 (d × d × t, *J* = 17.1 Hz, *J* = 10.5 Hz, ^3^*J* = 5.7 Hz, 2H), 5.27 (d × d × t, *J* = 17.1 Hz, *J* = 1.5 Hz, *J* = 1.5 Hz, 2H), 5.18 (d × d × t, *J* = 17.1 Hz, *J* = 1.8 Hz, *J* = 1.2 Hz, 2H), 4.02 (d × t, *J* = 5.7 Hz, *J* = 1.5 Hz, 4H), 3.68–3.58 (m, 16H). DART HRMS (m/z) calcd for C_14_H_27_O_5_ [M+H]^+^: 275.1853; found 275.1851.

#### EMP-adamantane (EMP-Ad)

The synthesis of 2-ethylsulfanylthiocarbonylsulfanyl-2-methyl-propionic acid (EMP) was performed as previously reported (Lai et al., 2002; Convertine et al., 2006). To a 25 mL round bottom flask was added EMP (179.5 mg, 0.80 mmol), 1-adamantane methanol (159.6 mg, 0.96 mmol), 4-dimethylaminopyridine (DMAP) (19.5 mg, 0.16 mmol) and 5 mL DCM. After all reagents were dissolved, *N*,*N*′-dicyclohexylcarbodiimide (DCC) (247.6 mg, 1.20 mmol) was added in one portion. The reaction mixture was stirred at room temperature overnight. The precipitate was filtered and the solvent was removed under vacuum. The crude product was purified by silica gel column chromatography, eluted with 30% DCM in hexanes (TLC *R*_*f*_ = 0.30, yellow or stained with KMnO_4_ solution) to afford 210 mg **5** as a bright yellow solid (yield 70.5%).

^1^H NMR (300 MHz, CDCl_3_) δ 3.60 (s, 2H), 3.25 (q, *J* = 7.5 Hz, 2H), 1.93 (s, 3H), 1.71–1.60 (m, 12H), 1.49 (d, *J* = 2.4 Hz, 6H), 1.28 (d, *J* = 7.5 Hz, 3H). ESI HRMS (m/z) calcd for C_18_H_28_O_2_S_3_ [M+H]^+^: 373.1343; found 373.1324.

#### PNIPAM-Ad

*N*-isopropyl acrylamide (NIPAM) was freshly purified by sublimation and azobisisobutyronitrile (AIBN) was recrystallized twice from ethanol. In polymerization, NIPAM (1.09 g, 9.6 mmol), EMP-Ad (29.8 mg, 0.080 mmol), AIBN (2.63 mg, 0.016 mmol) and 4.8 mL acetonitrile were added to a 25 mL Schlenk tube. The reaction mixture was subjected to 3 cycles of freeze-pump-thaw to degas oxygen. The reaction was heated at 60°C for 4 h, after which polymers were recovered by precipitation in diethyl ether. The molar mass of the obtained polymer was 7.8 kg/mol (from GPC, dispersity *ĐH* = 1.05), 7.4 kg/mol (from ^1^H NMR endgroup analysis) and 7.0 kg/mol (from MALDI-TOF MS), respectively.

### Extending protein chains using different cysteine coupling chemistries (including branching reactions)

#### Thiol-maleimide

Protein Cys-P_4_-Cys was dissolved in denaturing buffer (8 M urea and 100 mM phosphate, pH 8.0) to reach a concentration of 10% (w/v). Tris(2-carboxyethyl)phosphine (TCEP) (20 eq.) was added to the solution and the pH was adjusted to 7.5. Bismaleimide **1a** or **1b** was dissolved in DMF, and was added to the solution (1 eq.). The reaction was stirred at room temperature for 3 days, and it was then dialyzed against MilliQ water and lyophilized. The long reaction time was chosen based on experimental results to maximize endgroup conversion and entanglement in gels.

#### Thiol-bromomaleimide

Protein Cys-P_4_-Cys was dissolved in denaturing buffer (8 M urea and 100 mM phosphate, pH 8.0) to reach a concentration of 10% (w/v). TCEP (20 eq.) was added to the solution and the pH was adjusted to 6.2. βCD functionalized dibromomaleimide **2** was dissolved in DMSO, and was added to the solution (1 eq.). The reaction was stirred at 4°C for 7 days, and it was then dialyzed against MilliQ water and lyophilized.

#### Thiol-ene

Protein Cys-P_4_-Cys was dissolved in denaturing buffer (8 M urea and 100 mM phosphate, pH 8.0) to reach a concentration of 10% (w/v). TCEP (20 eq.) was added to the solution. The mixture was left to stir at room temperature for 4 h, and it was then dialyzed against MilliQ water and lyophilized. After reduction, >99% of the proteins were in the monomeric state, assessed by SDS-PAGE (Supplementary Figure 2). Reduced proteins were hydrated in 100 mM sodium phosphate buffer (pH 7.6), and mixed with **3** (1 eq.) and VA-044 (0.2 eq.). The final concentration was adjusted to 20% (w/v). Hydrogel samples were loaded on the rheometer after hydration for 2 days. The reaction was triggered by heating at 60°C for 3 h. The moduli were monitored at 25°C to ensure that the steady state was reached before measurement.

In branching reactions, the trifunctional crosslinker 2,4,6-triallyloxy-1,3,5-triazine **6** was used in place of the bifunctional crosslinker **3**. The total number of alkene groups was kept in 1:1 molar ratio to thiol groups, while the amount of **6** was varied from 0–100% of the total mole fraction of alkene-containing oligomer.

### Rheology

Rheology experiments were performed on Anton Paar MCR 301, 501, and 702 rheometers using a cone and plate geometry (25 mm diameter, 1° cone and 50 μm truncation gap) or a parallel plate geometry (10 mm diameter and 300 μm gap, only for measurement with βCD-functionalized protein hydrogels). The quantitative measurement of hydrogel mechanics was not affected by the choice of rheometers. Lyophilized proteins were hydrated in 100 mM sodium phosphate buffer (pH 7.6) to a final concentration of 20% (w/v). Hydrogels were kept at 4°C for 2 days to allow complete hydration. In order to minimize dehydration during measurement, the edges of hydrogel samples were coated with mineral oil. After loading onto the rheometer, samples were heated from 25°C to 90°C and cooled to 25°C at 5°C/min. Unfolding of the coiled-coil domains at high temperatures allowed rapid stress relaxation within the gel to eliminate any shear history, but no sol-gel transition was observed over this temperature range (Supplementary Figure 1). Frequency sweep measurements were performed at 1% strain in the linear viscoelastic regime (LVE). In creep experiments, a 25 Pa load was exerted on hydrogels for 2 h, and the load was removed to monitor the recovery behavior for 2 h. The creep-recovery experiment was repeated at 50 Pa load to ensure the deformation was independent of the applied load.

## Results and discussion

### Comparison of various chemistries on the chain extension reaction

**Scheme 1:**
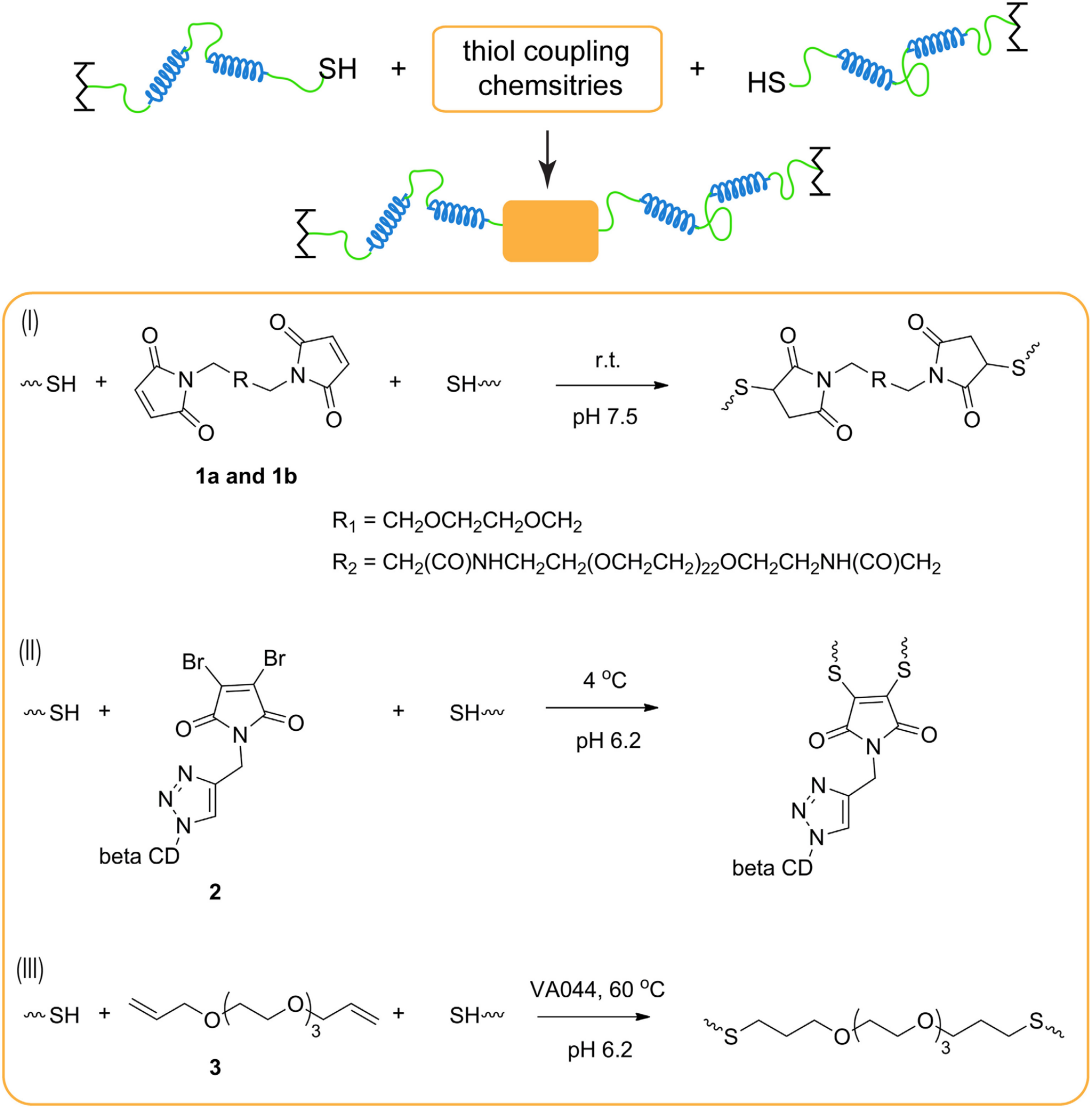
**Cysteine coupling chemistries to extend protein chains. (I)** Thiol-maleimide conjugation; **(II)** thiol-dibromomaleimide conjugation; and **(III)** thiol-ene chick chemistry.

**Figure 1:**
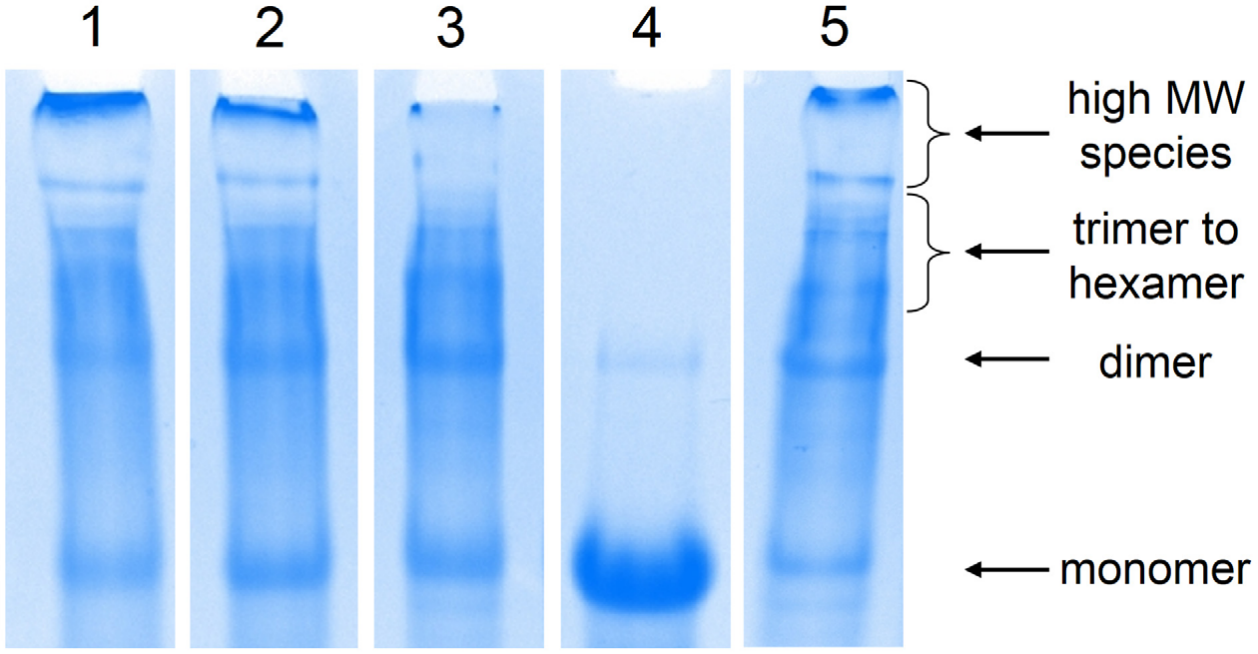
**Analysis of the chain extension products by SDS-PAGE**. Lane 1: thiol-maleimide with 1a; lane 2: thiol-maleimide with 1b; lane 3: thiol-dibromomaleimide; lane 4: reduction of chain-extended proteins by BME; lane 5: thiol-ene. The abundance of high molecular weight species in thiol-ene and thiol-maleimide corresponds to their higher entanglement plateau.

Chain extension of cysteine end-capped proteins can be achieved by applying thiol-maleimide, thiol-bromomaleimide and thiol-ene coupling chemistries under appropriate reaction conditions (Scheme 1 and Figure 1). All chemistries require reduction of existing disulfides to recover reactive cysteine residues. In order to achieve significant changes in mechanical properties, the conversion of these macromolecular polycondensation reactions must be relatively high to produce proteins of high molecular weights. In practice, this is achieved by controlling stoichiometry of the reacting species. A theoretical full conversion of endgroups can only be obtained when the ratio of cysteines to alkenes is 1:1. Running the reaction for extended periods is also useful to reach high conversions.

Each chemistry offers its own distinct advantages and disadvantages for chain extension. In the thiol-maleimide reaction, pH control is critical to minimize amine-maleimide coupling while achieving fast conjugation (Hermanson, 2008). In addition, dimaleimide reagents are commercially available with varying distance between the two maleimide groups, and this spacer length may affect the polycondensation reaction due to their subtle differences in solubility or by altering the propensity to form bridges or primary loops (Dutton et al., 1994). To study the effect of the distance between two conjugation sites, bismaleimide with two different oligo ethylene glycol spacer lengths **1a** and **1b** are used to conduct chain extension. It is found that the spacer length does not greatly affect the chain extension when the coupling reagents can be solubilized and homogeneously dispersed in the reaction.

As for thiol-dibromomaleimide coupling, controlling the hydrolysis of the maleimide ring, especially the monothio adduct, is crucial to attain significant chain extension. If hydrolysis happens on the dithio adduct, transformation from maleimide to maleamic acid does not affect chain extension, and it only results in loss of the reversibility of the thiomaleimide adduct (Supplementary Scheme 1). In contrast, hydrolysis of the monothio adduct can potentially limit the conversion, as the number of reactive bromomaleimide functional group decreases (Supplementary Scheme 1). Maleimide hydrolysis can be regulated in many ways, including changing temperature, pH (Ryan et al., 2011) and the electron density distribution in the maleimide structure (Nathani et al., 2013). In our hands, the first two parameters are optimized: a low temperature (4°C) and a slightly acidic buffer condition (pH 6.2) are chosen to be the reaction condition, under which moderate chain extension is achieved. After chain extension, the protein is exposed to 1000-fold excess BME, and ~ 98% of the proteins are converted to the monomeric state (Figure 1), which demonstrates the reversibility of the dithiomaleimide adduct.

In the thiol-ene coupling strategy, bis allyl compound **3** is chosen as the chain extender to prevent homopolymerization of alkenes that can occur when (meth)acrylate groups are used (Hoyle and Bowman, 2010), and water soluble VA-044 is selected as the thermal initiator for its low decomposition temperature. As the thiol-ene reaction is tolerant of oxygen (Hoyle et al., 2010), no cumbersome degassing procedure is required, enabling its convenient use in applications. Upon heating at 60°C for 3 h, a large fraction of high-molecular-weight proteins is formed. While thermally initiated chain extension is used here in order to ensure homogeneous reaction through a concentrated solution, it is also possible to perform photoinitiated chain extension using this chemistry, especially in occasions when spatial and temporal control of gel mechanics is required. TCEP needs to be removed prior to the thiol-ene reaction; otherwise the degree of chain extension is fairly low (Supplementary Figure 2). The deleterious effect of TCEP may be due to the desulfurization of cysteines catalyzed by TCEP during free-radical-based reactions, an effect that has been observed under similar reaction conditions (Wan and Danishefsky, 2007).

**Figure 2:**
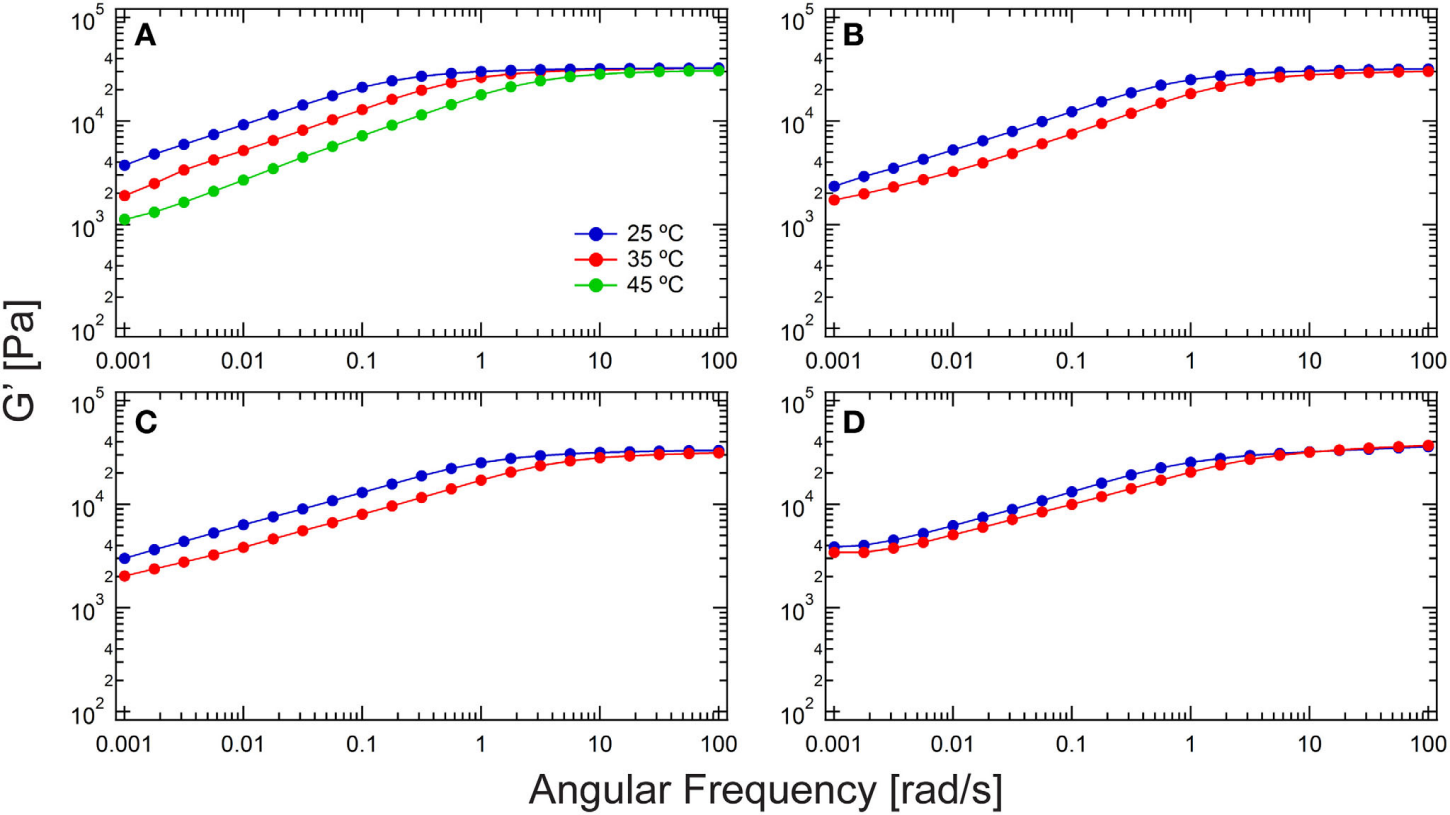
**Comparison of rheological frequency sweeps of chain extended hydrogels from various chemistries. (A)** Thiol-maleimide with 1a; **(B)** thiol-maleimide with 1b; **(C)** thiol-dibromomaleimide; and **(D)** thiol-ene. The plateau modulus in the low frequency regime down to 0.001 rad/s is the entanglement plateau. The frequency spectrum shifts to the high-frequency end at elevated temperatures.

Cysteine coupling chemistries exhibit different performances in the chain extension reaction, and the differences in the molecular weight distribution of the chain-extended proteins result in varying extents of entanglement in the protein hydrogels. As shown in Figure 2, hydrogels prepared *via* different chemistries all show an entanglement plateau modulus in the low-frequency regime. The chain molar mass distributions extracted from gels in Figure 1 do not fit well to the Jacobson-Stockmayer distribution, likely because of challenges in controlling stoichiometry of reagents in small samples and because of difficulty in quantitatively separating the large high molar mass band, which contains a mix of proteins with a molar mass of ca. 380 kg/mol and above (6-mers and above). Therefore, the chain extension and the entanglement effect is quantitatively analyzed by the entanglement plateau moduli in the low frequency regime. The apparent entanglement molecular weight *M*_*e*_ can be estimated as (Larson et al., 2003)

$$M_{e}=\frac{4}{5}\frac{\rho \varphi RT}{G_{e}}$$

with ρ being the protein density, ϕ the protein volume fraction in the gel, *R* the gas constant, *T* the absolute temperature and *G*_*e*_ the entanglement plateau modulus. Although the entanglement plateau modulus should not depend on the molecular weight of the protein polymers in the high molecular weight limit, proteins formed *via* macromolecular polycondensation show very broad molecular weight distributions with a certain fraction of the protein chains below the critical entanglement length. These “small” proteins act as macromolecular diluents and lower the effective concentration of the entangled species, consistent with the concentrations investigated being in the transition from the sticky Rouse phase to the sticky reptation regime (Tang et al., 2014). Therefore, increasing chain molecular weight, particularly the low molar mass tail, can increase *M*_*e*_. Consequently, differences in the degree of chain extension will yield differences in the plateau modulus of gels.

**Figure 3:**
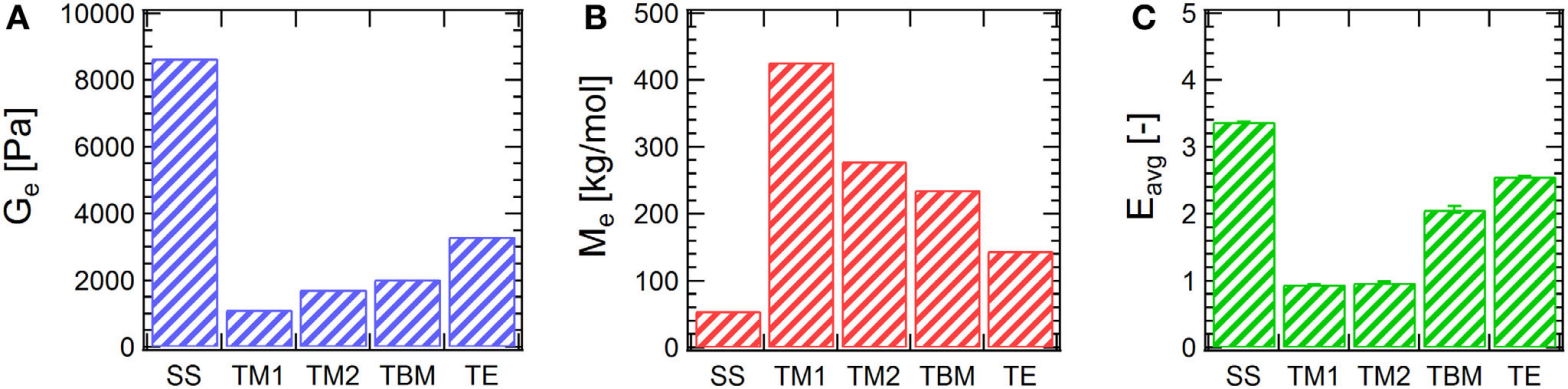
**Comparison of (A) entanglement plateau moduli, (B) the apparent entanglement molecular weights, and (C) the average entanglement densities**. SS: disulfide, data from Tang et al. (2014); TM1, thiol-maleimide with 1a; TM2, thiol-maleimide with 1b; TBM, thiol- dibromomaleimide; TE, thiol-ene. Error bars represent a 95% confidence interval.

Compared to disulfide bridging investigated previously (Tang et al., 2014), the three chemistries examined here show lower degrees of chain extension. *G*_*e*_ and *M*_*e*_ are compared in Figures 3A,B. For 20% (w/v) hydrogels, disulfide coupling leads to a *G*_*e*_ of around 8800 Pa, approximately 2–8 times larger than the *G*_*e*_'s from other chemistries. As a result, *M*_*e*_ is only 55 kg/mol in the disulfide coupling, even smaller than the molecular weight of a monomeric protein. The reasons for the lower entanglement molar masses in disulfide coupling are two-fold. First and most importantly, the stoichiometric imbalance between alkenes and thiols, due to inevitable experimental errors, set a practical limit of the functional group conversion in the A–A + B–B type macromolecular polycondensation that becomes more acute for chemistry performed on small samples. On the contrary, the disulfide bridging chemistry has no theoretical limit in the degree of chain extension due to stoichiometry since it is an A-A type polycondensation. Second, there are various side reactions in thiol-maleimide, thiol-bromomaleimide and thiol-ene coupling, which limit their ability to reach full conversion. As mentioned previously, the primary concern is that the occurrence of side reactions may reduce the availability of the reactive functional groups (e.g., maleimide hydrolysis) or cause uncontrolled chain coupling (e.g., amine-maleimide coupling). These side reactions further exacerbate challenges in controlling the stoichiometry of A and B reactive groups in the macromolecular polycondensation. *G*_*e*_not only provides clues to analyze the extent of entanglement, but also provides information on the effect of side reactions. The difference in *G*_*e*_might indicate that fewer side reactions occur in the thiol-ene coupling in the three chemistries examined in the study. Interestingly, although coupling with **2** only reaches moderate chain extension and side reactions might have the most detrimental effect, *G*_*e*_from thiol-bromomaleimde coupling is larger those from thiol-maleimide chemistries. It is hypothesized that the supramolecular association between βCD and amino acid residues forms weak and dynamic crosslink junctions (with equilibrium constant log*K* around 2–3) (Rekharsky and Inoue, 1998), thus increasing the modulus in the low-frequency regime.

Creep experiments also provides information to compare the entanglement effects in gels prepared by different chemistries. Here, entanglement density *E* is used to quantify the entanglement effect, a quantity defined as the number of entanglements per molecule, namely, *E* = *M*/*M*_*e*_ (Graessley, 2008). Strictly speaking, the definition above is used to describe the extent of entanglement of monodisperse polymers. Here the concept is borrowed to provide an estimate of the number-averaged entanglement density. In the low *E* limit (when *E* < 6), the average entanglement density in the chain extended protein mixtures can be calculated as

$$E_{avg}=2.5J_{e}^{0}G_{e}$$

where *J*^0^_*e*_ is the recoverable compliance, calculated as the extrapolated intercept from a linear fit in steady state of the *J-t* data (Supplementary Figure 3); *G*_*e*_ is the entanglement plateau modulus, as defined by Graessley (2008) differently than the definition by Larson et al. shown above by a factor of 4/5. As shown in Figure 3C, the low *E* values confirm the previous assumption that the entanglement density is not large. It is also found that the average entanglement densities of the chain-extended proteins gives the same trend as the entanglement plateau moduli in gels, namely, disulfide > thiol-ene > thiol-bromomaleimide > thiol-maleimide. For entangled gels prepared *via* thiol-maleimide coupling, *E*_*avg*_ is only approximately unity. On the contrary, *E*_*avg*_ is larger than 3 for gels prepared *via* disulfide bridging.

**Scheme 2:**
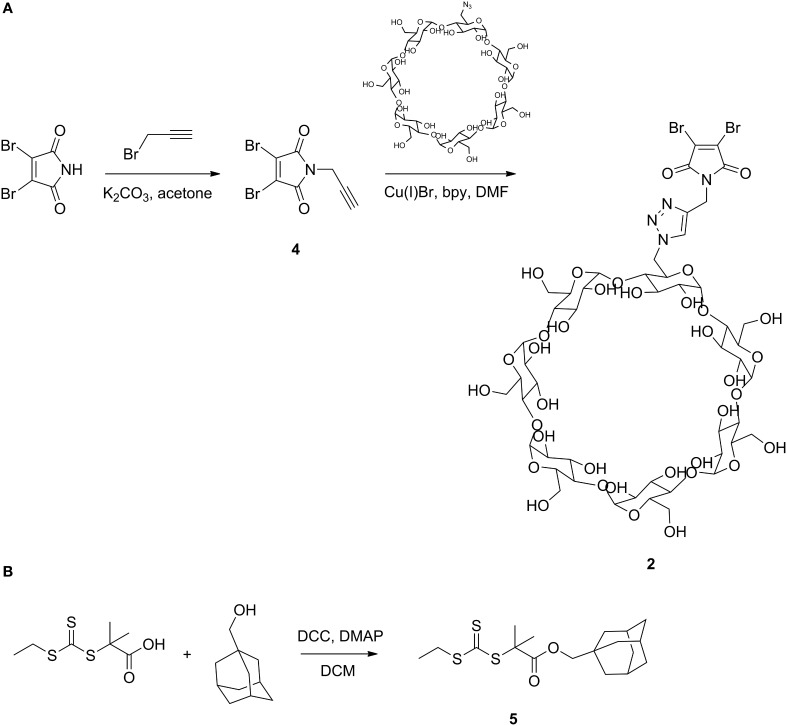
**Synthesis of dibromomaleimide functionalized βCD (A) and adamantane functionalized trithiocarbonate RAFT agent (B)**.

**Figure 4:**
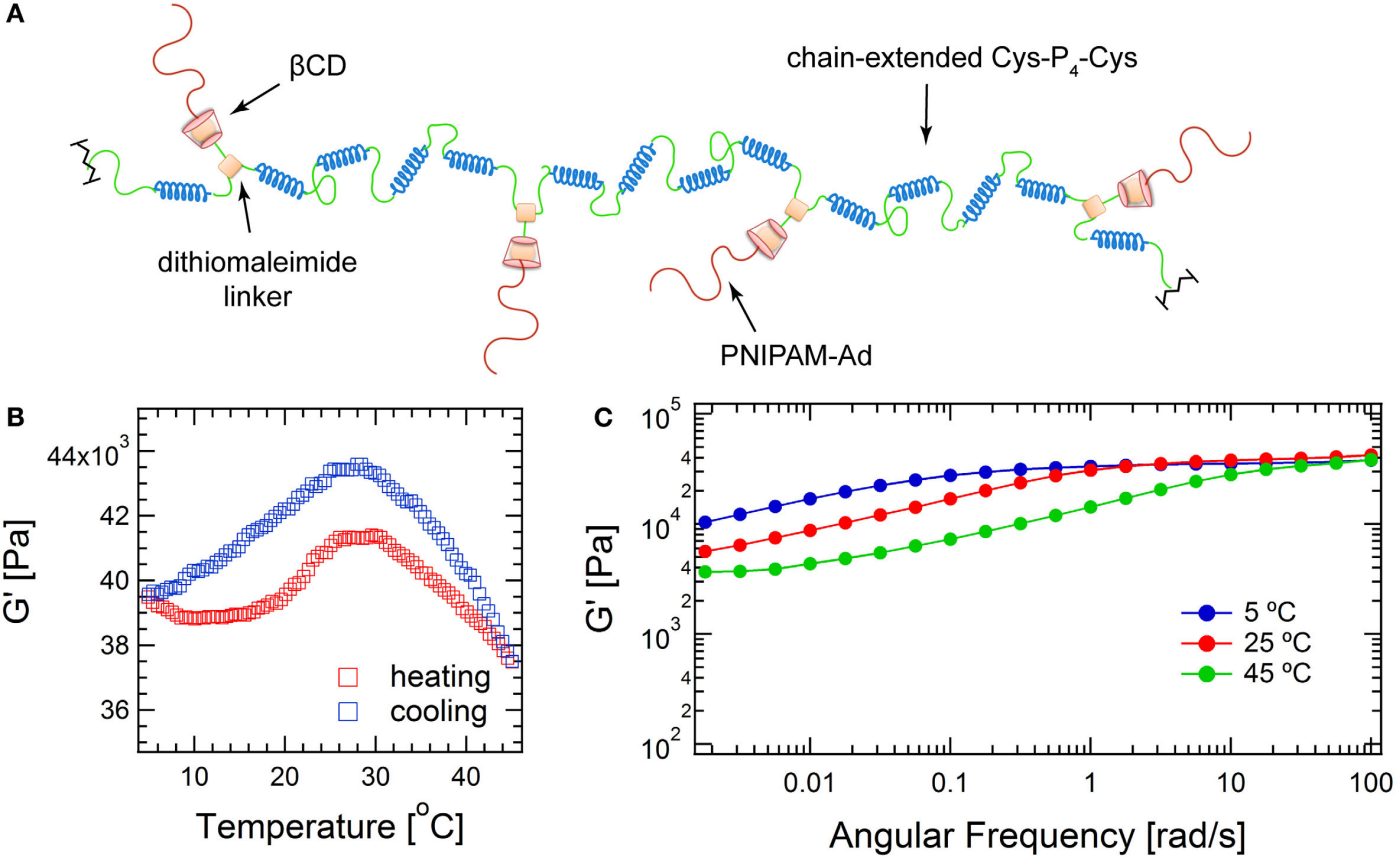
**Structure and mechanics of hybrid protein hydrogel. (A)** Illustration of the brush-like structure of PNIPAM-extended-P_4_ chimeric molecule formed *via* βCD-adamantane complexation; **(B)** Temperature sweep at 1°C/min heating/cooling rate; **(C)** Frequency sweep spectra.

Because dibromomaleimide can be readily functionalized, dibromomaleimide coupling provides a facile method of incorporating biological niches into the hydrogel network and embedding additional stimuli triggers to manipulate network structure. To illustrate this concept, dibromomaleimide is first derivatized with alkyne, and is further functionalized with βCD through copper-catalyzed azide-alkyne cycloaddition (CuAAC) to obtain **2** (Scheme 2). βCD is chosen because its internal cavity can host many small guest molecules, such as drugs, *via* hydrogen bonding and hydrophobic interactions (Davis and Brewster, 2004). The most widely used complexation pair is βCD-adamantane with an association constant *K*_*a*_ of about 5 × 10^4^ M^−1^ (Harries et al., 2005). This supramolecular association may offer modification sites to attach functionalities to the protein backbone. Here, the βCD-adamantane host-guest interaction is used to demonstrate the ability to add thermoreversible association to hydrogels. Adamantane end-capped monodisperse PNIPAM is synthesized by reversible addition-fragmentation chain transfer (RAFT) polymerization using trithiocarbonate **5** as the RAFT agent (Supplementary Figure 4), and the polymer's endgroup structure is confirmed by MALDI-TOF and NMR (Supplementary Figures 5, 6, respectively). Upon complexation, the hybrid structure is hypothesized to adopt a graft-like structure: the chain-extended protein serves as backbone while the thermoresponsive polymers PNIPAM graft as side chains (Figure 4). Under the experimental conditions, approximately 91.8% of the PNIPAM can be attached to the proteins (see calculation details in Supplementary Materials and Supplementary Figure 7). The high-frequency plateau modulus (*G* ' at 100 rad/s) of the hybrid gel is 10 kPa larger than the unmodified gel, and it increases moderately with increasing temperature, with a peak value at around 28°C. The entanglement modulus also increases from 2030 to 3670 Pa. The enhancement in the both moduli might originate from changes of protein architecture, and the formation of nanostructure within the gel due to segregation between the PNIPAM domain and the protein domain. Such microphase separation may even exist at low temperature, as enhancement in moduli is observed as low as 5°C. This observation is consistent with the previous finding that PNIPAM segregation takes place even when the polymers are highly solvated (Glassman and Olsen, 2013).

### Influence of branched architecture on the mechanical properties

**Figure 5:**
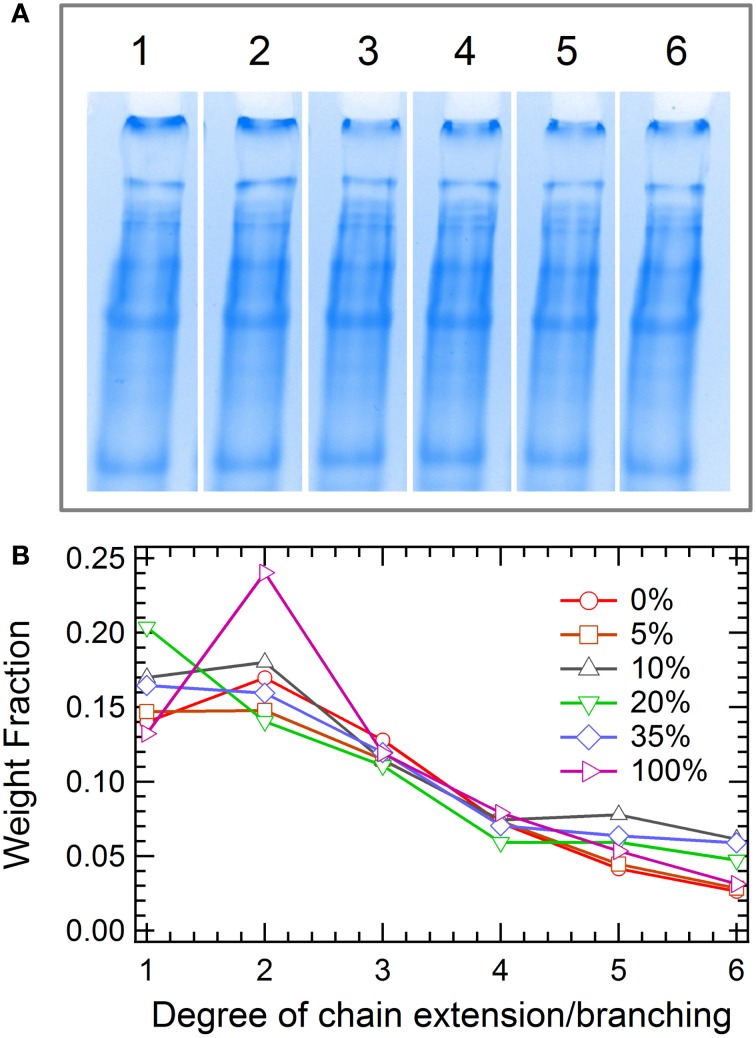
**Molecular weight distribution of proteins in the branching reaction. (A)** SDS-PAGE: The molar fractions of triene 6 in lanes 1–6 is 0, 5, 10, 20, 35, and 100%, respectively; **(B)** Weight fraction extracted by densitometry.

Branched polymers have attracted much interest for fundamental studies and industrial applications due to their distinct flow behaviors compared to their linear analogs (Dealy and Wissbrun, 1990; Janzen and Colby, 1999; Wood-Adams and Costeux, 2001; Lohse et al., 2002; Graessley, 2008; Wang et al., 2010b). However, few reports characterize the synthesis or properties of branched proteins, using either chemical methods or cellular machinery (Zhang et al., 2013). Here, branched proteins are synthesized with the use of multifunctional crosslinkers in the chain extension reaction, demonstrating an important means to control the mechanical properties of protein gels by modulating the chain structure. The effect of branching is examined by varying the fraction of the trifunctional crosslinker (2,4,6-triallyloxy-1,3,5-triazine, **6**) from 0 to 100% in the total alkenes (note that the ratio of total alkenes to thiols is kept 1:1). The hydrogels are prepared by thiol-ene click chemistry *via* thermal initiation. Similar to chain extension, all branching reactions are found to reach high endgroup conversion, yielding significant fractions of high molar mass proteins from SDS-PAGE (Figure 5). Significant differences in molecular weight distribution of proteins among the 6 reactions examined cannot be measured by gel electrophoresis. An exception occurs at 100% triene, where a larger fraction of low molar mass protein is present, suggesting that the endgroup conversion might be lower than those in other cases. All protein hydrogels are able to be dissolved in 6 M urea buffer post reaction, confirming the hypothesis that addition of **6** only results in branching and not crosslinking, even with 100% triene crosslinker. The gel point under each reaction condition can be calculated using the Carothers equation or the Flory-Stockmayer theory (see calculation details in Supplementary Materials and Supplementary Figure 8), which shows great dependence on the triene composition. The solubility of gels in urea suggests that the conversion of the endgroups is below the gel point in all cases.

The mechanical properties of hydrogels are influenced by the branched structure of the proteins. To better compare the entire relaxation spectra with different fractions of **6**, rheology data from creep experiments are converted to the dynamic compliance *J*′ and *J*″ using a Fourier transform (Ferry, 1980),

$$\begin{array}{l} J^{\prime }\left(\omega \right)=J_{e}^{0}-\omega \int_{0}^{\omega }\left[J_{e}^{0}-J\left(t\right)+t/\eta_{0}\right]sin\omega tdt\\ J^{\prime \prime }\left(\omega \right)=1/\omega \eta_{0}+\omega \int_{0}^{\omega }\left[J_{e}^{0}-J\left(t\right)+t/\eta_{0}\right]cos\omega tdt \end{array}$$

where *J*^0^_*e*_ is the recoverable compliance, *ω* the angular frequency, η_0_ the zero-shear-rate viscosity, *t* the experiment time. *J*^0^_*e*_ and η_0_ are the intercept and the inverse of the slope, respectively, from a linear fit in steady-state phase of creep. The dynamic compliances are further translated into dynamic moduli *G*′ and *G*″,

$$\begin{array}{l} G^{\prime }=J^{\prime }/\left(J^{\prime }{}^{2}+J^{\prime \prime }{}^{2}\right)\\ G^{\prime \prime }=J^{\prime \prime }/\left(J^{\prime }{}^{2}+J^{\prime \prime }{}^{2}\right) \end{array}$$

**Figure 6:**
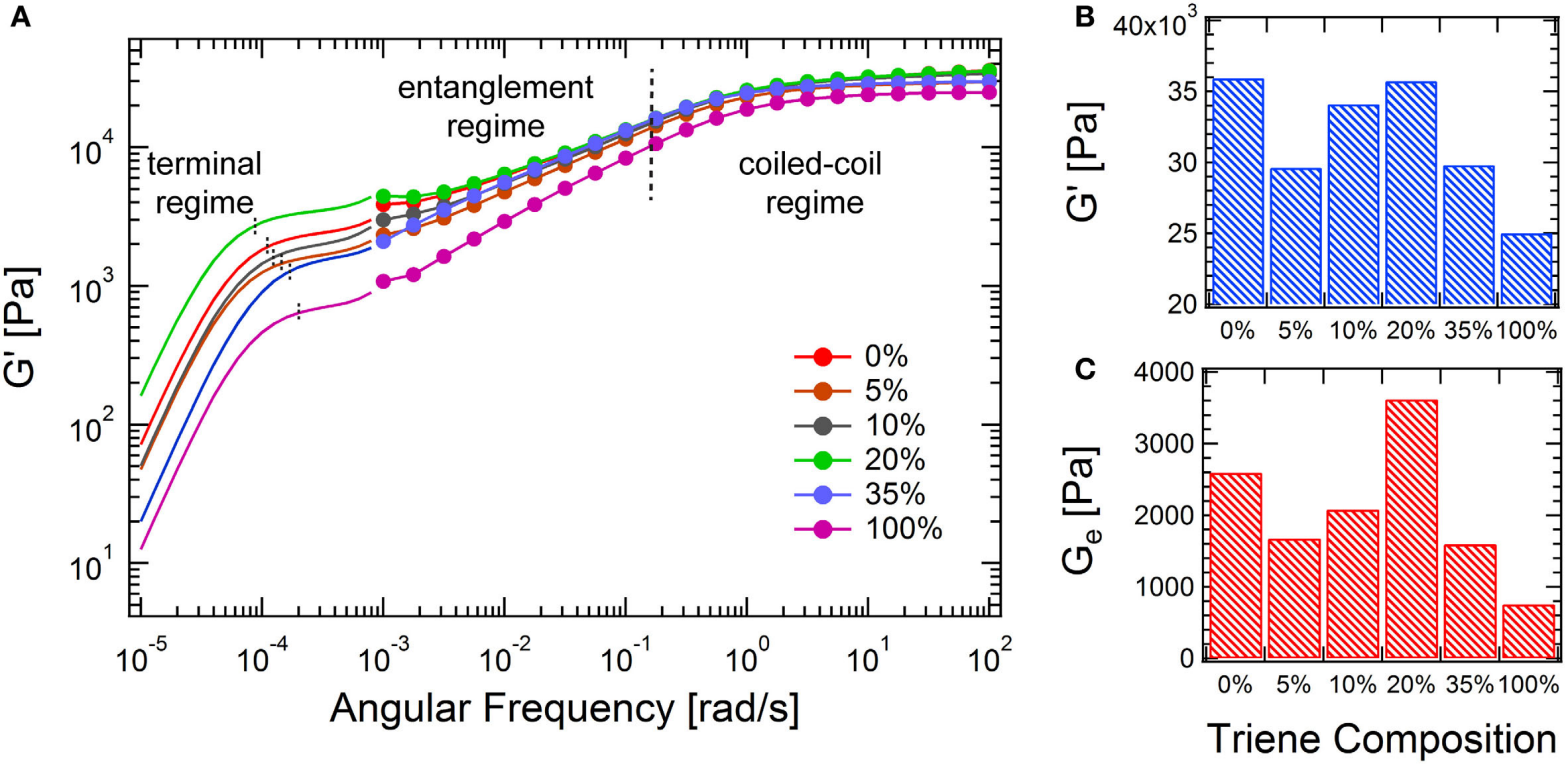
**Comparison of the mechanical properties of branched hydrogels at different triene compositions. (A)** Master curves at 25°C from oscillatory shear and creep experiments; **(B)** comparison of the high-frequency moduli; **(C)** comparison of the entanglement moduli, determined from the converted low-frequency plateau.

The combined master curves are shown in Figure 6, which can be divided into three different regimes according to the relaxations present in the gels (from fast to slow): the coiled-coil regime, the entanglement regime and the terminal regime. With addition of a small amount of **6** (5 and 10%), both the high-frequency modulus (*G*′_∞_) and the entanglement modulus (*G*_*e*_) decrease. This suggests that adding a small amount of crosslinker only causes short chain branching and small drops in the backbone length of the chain-extended proteins, since this would result in a decrease in the total number of entanglements. However, at 20% triene, both *G*′_∞_ and *G*_*e*_reach their peak values, where the number density of the branch points and the branch length reach an optimal combination. In this case, the backbone length of the protein is likely to be comparable to the linear case, as the molecular weight distributions from SDS-PAGE of these two cases are not distinguishable, but the branched structure provides extra topological interactions. The introduced branches retard the reptation of the entire molecule, as it is only after arm retraction that the reptation of the chain-extended backbone is allowed to happen (Mcleish, 2002). Although the coiled-coil relaxation is only slightly affected by the molecule topology (see Supplementary Figures 9, 10), the reptation regime broadens in the frequency spectrum and extends toward the low frequency regime. This is consistent with the observation of randomly branched polymers in the melt state (Mcleish, 2002). Further increase of the triene composition leads to decreases in both *G*'_∞_ and *G*_*e*_. This is hypothesized to originate from the decreased engroup conversion and the formation of hyperbranched molecular structures. The densely packed branches impose permanent topological barriers for different chains to entangle, which leads to much lower solution viscosities (Voit and Lederer, 2009). Recently, the viscoelasticity and the dynamic relaxation of the synthetic hydrogels is found to be essential to mimic the complex biological tissues (Mckinnon et al., 2013). The presented branching reaction in this study establishes a facile approach of tuning the network mechanics over the entire frequency window, especially in the long time relaxation, which can be useful in mimicking the properties of natural tissues.

## Conclusion

Under appropriate conditions, thiol-maleimide, thiol-bromo-maleimide and thiol-ene coupling can all result in significant chain extension. The differences in chemistries' reactivity and side reactions may cause variations in the molecular weight and its distribution of chain-extended proteins and the topological entanglement effect in gels. While thiol-maleimide conjugation is the most common and the easiest to implement, thiol-ene click chemistry can achieve high endgroup conversion fairly rapidly. Thiol-bromomaleimide shows its potential in reversibly modifying proteins, and using functionalized dibromomaleimide as a chain extender allows further control of hydrogels' properties by incorporating side chain functionalities into the protein architecture. Here, thermoresponsive changes in mechanical properties of gels are demonstrated with PNIPAM grafts. Lastly, branched proteins are prepared in A_2_ + B_2_/B_3_ mixed type reactions. The entanglement plateau modulus is increased when branches are long enough to enhance topological constraints yet the added branches do not sacrifice the backbone length. In conclusion, the structure of engineered proteins and their assembly behaviors can be easily modified with the use of different chemistries, which presents a rich toolbox to tailor the structure-properties of protein materials in various applications.

### Conflict of interest statement

The authors declare that the research was conducted in the absence of any commercial or financial relationships that could be construed as a potential conflict of interest.
