# Supplementary material for: Controlling topological entanglement in engineered protein hydrogels with a variety of thiol coupling chemistries
Source: Front Chem. 2014 May 14;2:23. doi: 10.3389/fchem.2014.00023 (PMC4030145; doi:10.3389/fchem.2014.00023)
Supplement: Supplementary file 1 [file Data_Sheet_1.PDF]

Supplementary Material
10.3389/fchem.2014.00023

Supplementary Material

Controlling Topological Entanglement in Engineered Protein Hydrogels with a Variety of Thiol Coupling Chemistries

Shengchang Tang[1]**, and Bradley D. Olsen**[1]*****

[1]Department of Chemical Engineering, Massachusetts Institute of Technology, Cambridge, MA, USA

*** Correspondence:** B.D. Olsen, Department of Chemical Engineering, Massachusetts Institute of Technology, 77 Massachusetts Avenue, Cambridge, MA, 02139, USA.
bdolsen@mit.edu

1. Supplementary Data

1.1. Estimation of the theoretical fraction of PNIPAM associated with chain-extended proteins

Assuming βCD is all attached to the proteins, the concentration of βCD in the chain-extended protein hydrogel can be calculated as

$$c = \frac{n}{V} = \frac{\frac{200\ (mg)}{63000\ (g/mol)}}{1000 + \frac{200}{1.3}\ (\mu L)} = 2.75(mM)$$

And 1 eq. of adamantane functionalized PNIPAM is used to mix with the chain-extended proteins. The association constant can be expressed as

$$\boldsymbol{K_a = \frac{[Complex]}{[CD][PNIPAM - Ad]} = \frac{1 - x}{cx^2} = 5 \times 10^4\ (M^{-1})}$$

where x is the fraction of the unassociated βCD and adamantane. The positive root to the equation gives the physical fraction of free βCD and adamantane, that is, 0.082. Therefore, about 91.8% of the PNIPAM is expected to participate in associations with proteins.

The calculation above assumes that the chemical modification does not result in great changes in association constant, which has been shown in literature.[1-5] Even if the association constant decreases by an order of magnitude, the complexation remains to be strong and more than 76% of the βCD and adamantane participate into the formation of inclusion complex (see Supplementary Figure 7).

1.2. Calculation of the gel points using the Carothers Equation and the Flory-Stockmayer theory.

From the Carothers equation:

Assuming the molar fraction of triene **6** in the total alkenes is α, the average functionality of the reaction mixture is

$$f_{avg} = \frac{1 \times 2 + (1-\alpha) \times 2 + \alpha \times 3}{1 + (1-\alpha) + \alpha} = \frac{4+\alpha}{2}$$

Thus the gel point is calculated to be

$$p_c = \frac{2}{f_{avg}} = \frac{4}{4+\alpha}$$

From the Flory-Stockmayer theory:

The gel point p_c approaching the limit $\bar{X}_w \rightarrow \infty$ is predicted to be

$$p_C = \frac{1}{\left[\left(f_{w,A}-1\right)\left(f_{w,B}-1\right)\right]^{1/2}}$$

where $f_{w,A}$ and $f_{w,B}$ are the weight average functionalities of groups A (thiols) and B (alkenes). In the branching reactions,

$$f_{w,A} = 2$$

$$f_{w,B} = \frac{2^2(1-\alpha) + 3^2\alpha}{2(1-\alpha) + 3\alpha} = \frac{4+5\alpha}{2+\alpha}$$

The gel point is

$$p_C = \frac{1}{\left[\left(f_{w,A}-1\right)\left(f_{w,B}-1\right)\right]^{1/2}} = \frac{1}{\left(\frac{4+5\alpha}{2+\alpha}-1\right)^{1/2}} = \left(\frac{2+\alpha}{2+4\alpha}\right)^{1/2}$$

The results are plotted in Supplementary Figure 8.

2. Supplementary Figures

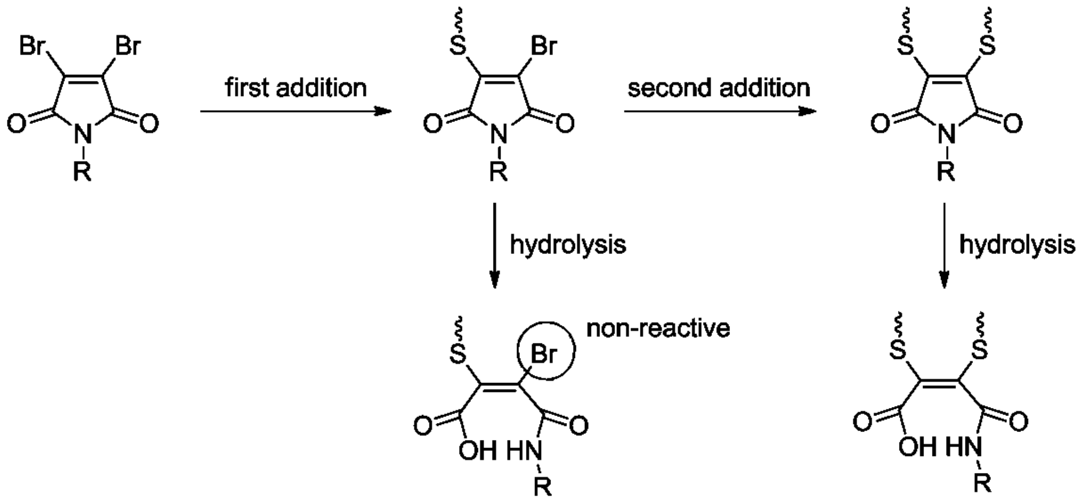

Supplementary Scheme 1. Hydrolysis of maleimide during the chain extension reaction via thiol-dibromomaleimide coupling.

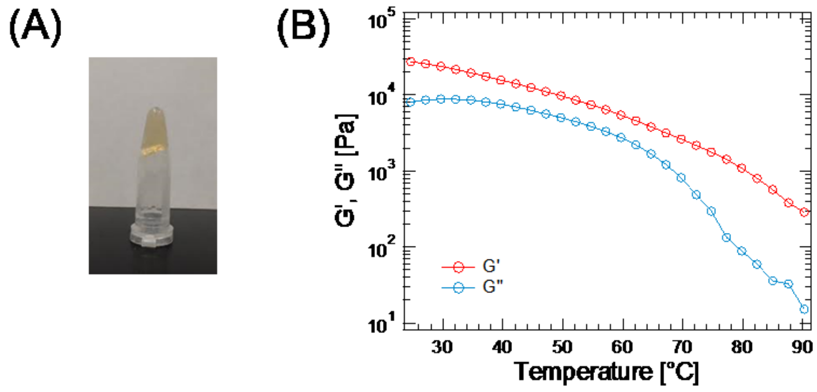

Supplementary Figure 1. (A) Inversion test showing the entangled hydrogel remains in the gel phase after holding at 100 °C for 1 min. (B) Representative temperature sweep of entangled protein hydrogels at 20% (w/v) concentration, measured at 1 rad/s and 1% strain. Both of the tests show that no sol-gel transition is observed in the experimental temperature window in the entangled hydrogel.

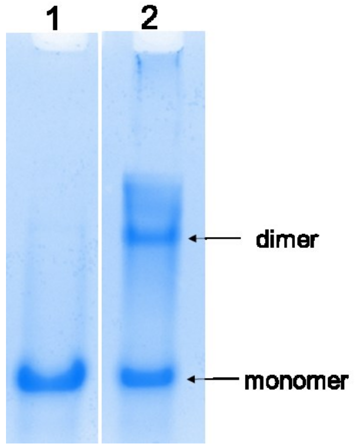

Supplementary Figure 2. Lane 1: SDS-PAGE of proteins after reduction by TCEP. More than 99% of the proteins are in monomeric state determined from densitometry. Lane 2: SDS-PAGE of thiol-ene chain extension with the presence of TCEP. Only a low degree of chain extension is reached.

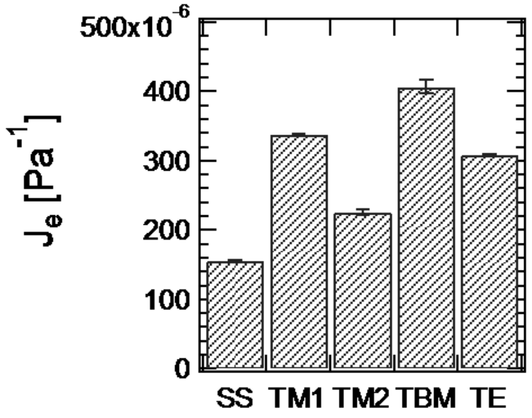

Supplementary Figure 3. Comparison of recoverable compliances in entangled hydrogels. SS: disulfide; TM1: thiol-maleimide with 1a; TM2: thiol-maleimide with 1b; TBM: thiol-dibromomaleimide; TE: thiol-ene.

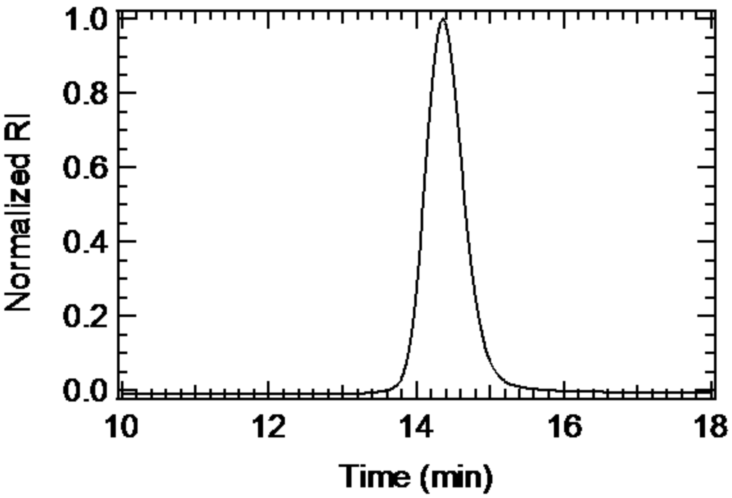

Supplementary Figure 4. GPC trace of PNIPAM-Ad. The number average molecular weight M_n = 7.8k and dispersity $Đ$ = 1.05.

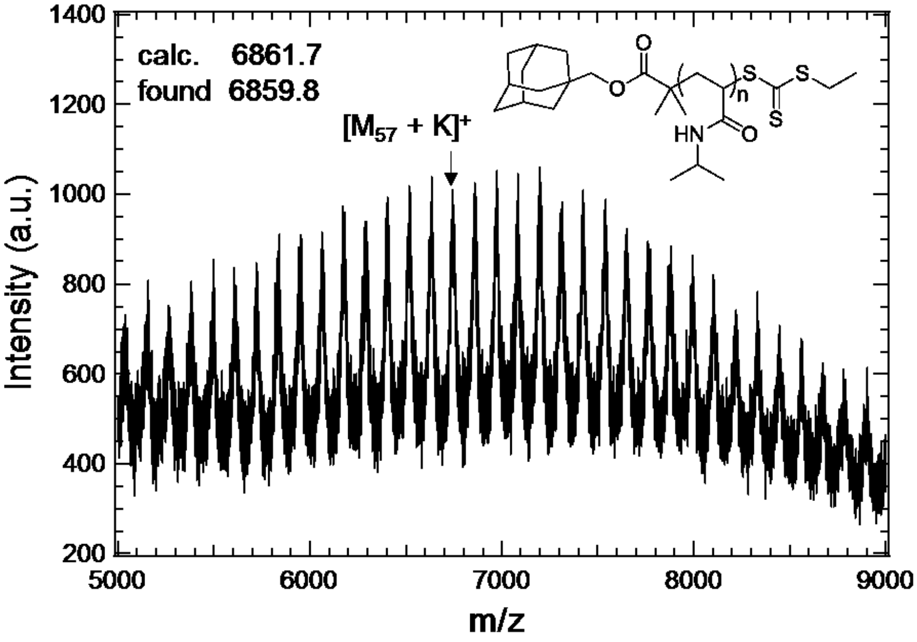

Supplementary Figure 5. MALDI-TOF spectrum of PNIPAM-Ad confirming the endgroup structure after polymerization.

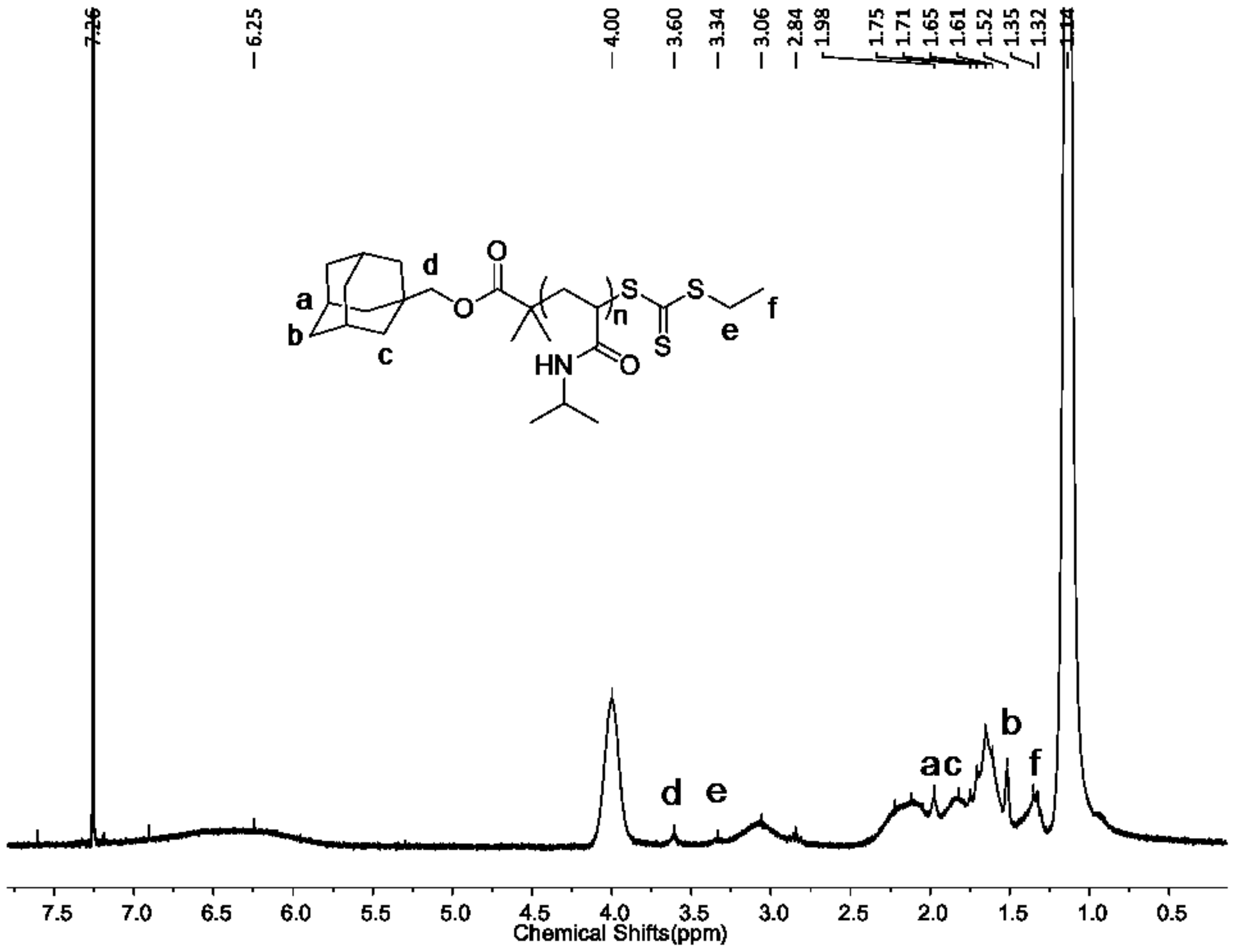

Supplementary Figure 6. NMR spectrum of PNIPAM-Ad confirm the presence of the endgroup post polymerization.

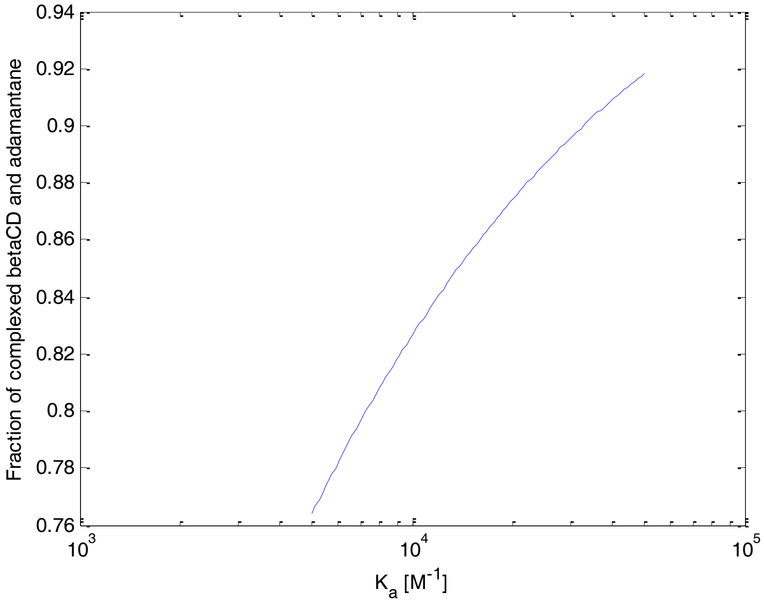

Supplementary Figure 7. Effect of K_a on the concentration of associating β-CD and adamantane.

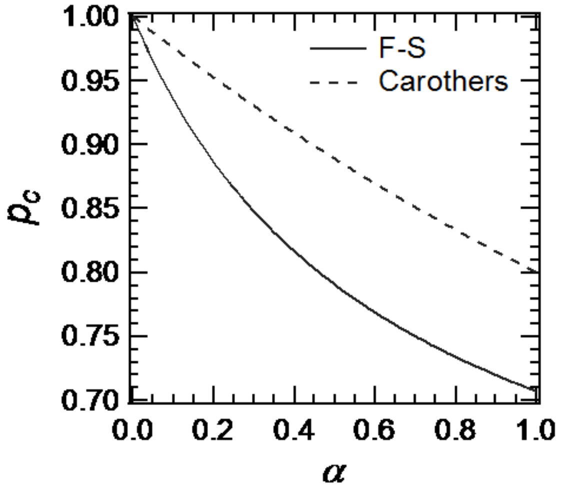

Supplementary Figure 8. The theoretical gel points from the Carothers and Flory-Stockmayer theories versus fraction of trifunctional crosslinker 6.

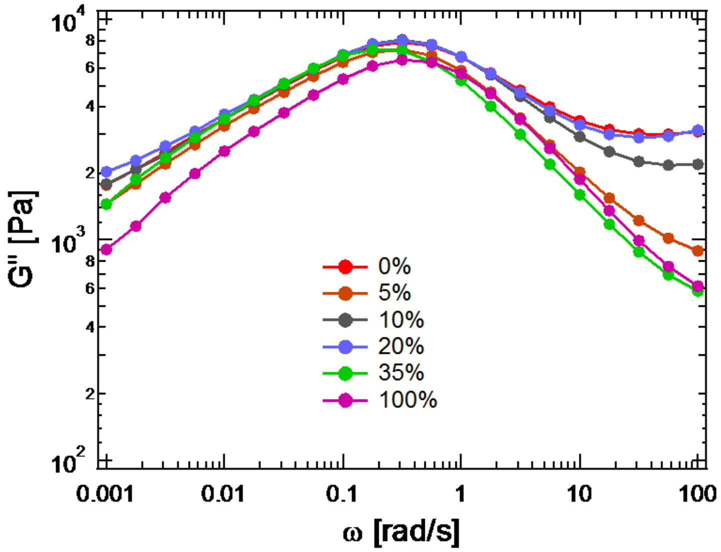

Supplementary Figure 9. Frequency spectra *G" ~ ω* at different triene compositions.

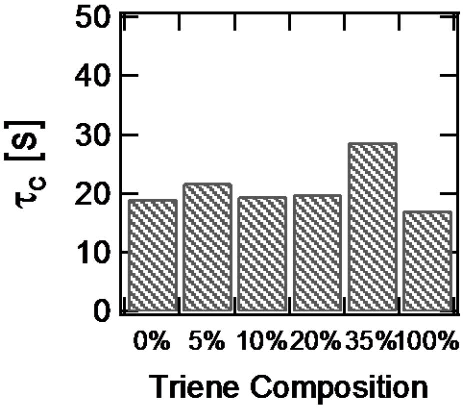

Supplementary Figure 10. The relaxation times of the coiled-coil domain in branched protein hydrogels at different triene compositions. The coiled-coil relaxation time is estimated as the inverse of the frequency where *G"* reach its maximum in the high-frequency regime, namely, $\tau_c = 2\pi/\omega$.

References:

1. Moers, C.; Nuhn, L.; Wissel, M.; Stangenberg, R.; Mondeshki, M.; Berger-Nicoletti, E.; Thomas, A.; Schaeffel, D.; Koynov, K.; Klapper, M.; Zentel, R.; Frey, H., *Macromolecules* **2013,** *46* (24), 9544-9553.
2. Osman, S. K.; Brandl, F. P.; Zayed, G. M.; Teßmar, J. K.; Göpferich, A. M., *Polymer* **2011,** *52* (21), 4806-4812.
3. Auzély-Velty, R.; Rinaudo, M., *Macromolecules* **2002,** *35* (21), 7955-7962.
4. Chiba, J.; Sakai, A.; Yamada, S.; Fujimoto, K.; Inouye, M., *Chemical Communications* **2013,** *49* (57), 6454-6456.
5. Weickenmeier, M.; Wenz, G., *Macromolecular Rapid Communications* **1996,** *17* (10), 731-736.
